# Investigation of the Use of a Bio-Derived Solvent for Non-Solvent-Induced Phase Separation (NIPS) Fabrication of Polysulfone Membranes

**DOI:** 10.3390/membranes8020023

**Published:** 2018-05-07

**Authors:** Xiaobo Dong, Amna Al-Jumaily, Isabel C. Escobar

**Affiliations:** Department of Chemical and Materials Engineering, University of Kentucky, Lexington, KY 40506, USA; xiaobo.dong@uky.edu (X.D.); amna.aljumaily@uky.edu (A.A.-J.)

**Keywords:** bio-derived solvent, non-solvent induced phase separation, membrane synthesis

## Abstract

Organic solvents, such as *N*-methyl-2-pyrrolidone (NMP) and dimethylacetamide (DMAc), have been traditionally used to fabricate polymeric membranes. These solvents may have a negative impact on the environment and human health; therefore, using renewable solvents derived from biomass is of great interest to make membrane fabrication sustainable. Methyl-5-(dimethylamino)-2-methyl-5-oxopentanoate (Rhodiasolv PolarClean) is a bio-derived, biodegradable, nonflammable and nonvolatile solvent. Polysulfone is a commonly used polymer to fabricate membranes due to its thermal stability, strong mechanical strength and good chemical resistance. From cloud point curves, PolarClean showed potential to be a solvent for polysulfone. Membranes prepared with PolarClean were investigated in terms of their morphology, porosity, water permeability and protein rejection, and were compared to membranes prepared with traditional solvents. The pores of polysulfone/PolarClean membranes were sponge-like, and the membranes displayed higher water flux values (176.0 ± 8.8 LMH) along with slightly higher solute rejection (99.0 ± 0.51%). On the other hand, PSf/DMAc membrane pores were finger-like with lower water flux (63.1 ± 12.4 LMH) and slightly lower solute rejection (96 ± 2.00%) when compared to PSf/PolarClean membranes.

## 1. Introduction

**Figure 1:**
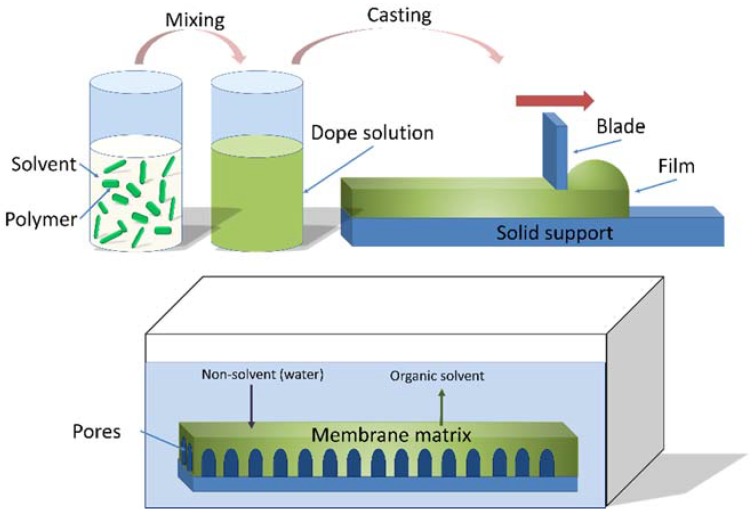
Non-solvent phase inversion casting process.

Membrane technology has proven to be effective in recent years due to its promising benefits such as reduced footprint, easy control and easy scale-up, simple operational parameters, high throughput and automation [1]. Asymmetric membranes typically consist of a porous support layer that provides mechanical strength and stability, and which is covered by a thin selective layer or film responsible for providing the membrane with separation capabilities [1,2,3]. Phase inversion usually includes non-solvent-induced phase separation (NIPS), thermally induced phase separation (TIPS), or a combination of both [2,4,5]. In the NIPS method, shown in Figure 1, a dope solution is prepared by dissolving a polymer in a solvent [6,7]. A membrane is then formed by the precipitation of the polymer in an anti-solvent bath, such as water. Briefly, through the immersion of a substrate in a coagulation bath, a solvent in the casting solution film is exchanged with a non-solvent in the precipitation media, and phase separation occurs. This process results in an asymmetric membrane with a dense top layer and a porous sublayer.

During membrane fabrication, large amounts of traditional organic solvents are used [8]. Solvents used in synthesis and post-synthesis steps can have a negative impact on operational safety, cost, the environment and human health [9,10,11]. Traditional solvents used for membrane preparation include dimethylformamide (DMF), *N*-methyl-2-pyrrolidone (NMP), dimethylacetamide (DMAc), dimethyl sulfoxide (DMSO), and tetrahydrofuran (THF). Some of these solvents are volatile and hazardous to the environment or living cells [12]. Solvents, such as cyclohexanes, DMAc, and DMSO are mutagenic and tumorigenic; acetone is highly flammable; and NMP is an irritant [12,13]. Acute effects of DMF include skin irritation and dizziness, while its long-term effects are known to cause birth defects [14]. Due to their hazardousness, solvents require specialized control measures. Therefore, the need for greener, sustainable chemicals has prompted a great amount of research into the processing of renewable feedstocks to obtain platform molecules and downstream end products. The annual global solvent market is in the order of 20 million metric tons and billions of dollars, and bio-based solvents consumption in Europe has been predicted to grow to one million metric tons by 2020 [15,16]. Using renewable solvents derived from biomass, which do not compete with food applications, satisfies both consumer and legislative demands with regards to sustainability. 

Therefore, solvents involved in the membrane manufacturing process are proposed to be replaced by greener alternatives. A green solvent is expected to be non-toxic, non-volatile, and derived from renewable sources [17]. In this research, a solvent produced from renewable sources, Methyl-5-(dimethylamino)-2-methyl-5-oxopentanoate (Rhodiasolv^®^ PolarClean, Solvay Novecare (Princeton, NJ, USA)), was used to replace the traditional solvents [18]. PolarClean is derived from the valorization of 2-methylglutaronitrile (MGN), a byproduct from the synthesis of Nylon 6,6 [19,20]. PolarClean has been previously shown to be a water-soluble, eco-friendly and biodegradable polar solvent with an excellent toxicological and eco-toxicological profile [19,21], and the major physicochemical properties of PolarClean are shown in Table 1 [12,21,22].

PolarClean has been previously investigated to cast polyvinylidene fluoride (PVDF) and polyethersulfone (PES) membranes [18,19,23,24]. Hassankiadeh et al. [18] used PolarClean as a green solvent to fabricate PVDF hollow fiber membranes via temperature induced phase separation (TIPS) process. Due to PolarClean’s high miscibility with water, Jung et al. [24] further investigated its NIPS effect on the membranes surface during TIPS process, along with the kinetics of the membrane formation process. Marino et al. [19] prepared PES ultrafiltration and microfiltration membranes using PolarClean using NIPS and vapor induced phase separation (VIPS) processes. However, the thermodynamics of polymer/solvent/non-solvent mixing and demixing processes when PolarClean is used as a NIPS solvent for the fabrication of polysulfone (PSf) membranes is unknown. Therefore, the research project described here first investigated the thermodynamics of mixing and demixing processes of PolarClean/PSf and compared them to NMP/PSf and DMAc/PSf. After mixing/demixing analysis (i.e., cloud point determination), DMAc was determined to be a more appropriate comparison for its greater similarity to PolarClean; subsequently, PolarClean was used to fabricate PSf membranes in a NIPS process and compared only to DMAc.

Polysulfone was chosen as the polymer to fabricate membranes due to its thermal stability, strong mechanical strength, good chemical resistance, and antifouling properties [25]. To dissolve PSf, NMP and DMAc were used as the traditional petroleum-based solvents since they are two of the most commonly used solvents in membrane fabrication and their performance has been studied for decades [25,26,27,28,29,30,31,32,33]. 

## 2. Experimental

### 2.1. Materials

Polysulfone (PSf, average Mw 35,000 by LS, average Mn 16,000 by MO, pellets) was purchased from Sigma-Aldrich (Saint Louis, MO, USA). Methyl-5-(dimethylamino)-2-methyl-d-oxopentanoate (Rhodiasolv^®^ PolarClean) was provided by Solvay Novecare (Princeton, NJ, USA). *N*,*N*-Dimethylacetamide (DMAc) was purchased from Tokyo Chemical Industry Co., Ltd. (Tokyo, Japan), and 1-Methyl-2-pyrrolidone (NMP, for peptide synthesis) was purchased from EMD Millipore Corporation (Burlington, MA, USA). Bovine Serum Albumin (BSA) was purchased from VWR Life Science (Radnor, PA, USA), and different sizes of polyethylene glycols (PEGs) were purchased from Alfa Aesar (Haverhill, MA, USA).

### 2.2. Thermodynamics

#### 2.2.1. Hansen Solubility Parameter Calculation

To choose the appropriate solvent for a polymer, the polymer must be soluble or easily dispersible in the specific solvent [27]. To select potentially compatible solvents, the relative energy difference (RED) is calculated using Equation (1):

RED = R_a_/R_o_ (1)

where *R_o_* is the radius of interaction of a Hansen solubility parameter sphere and *R_a_* is the solubility parameter distance between polymer (1) and solvent (2). *R_a_* can be calculated based on their individual Hansen solubility parameters ($\delta_{d}$ represents the dispersive force, $\delta_{p}$ represents the polar force and $\delta_{h}$ represents hydrogen bonding) using Equation (2) [34]:

(2) $$\mathrm{Ra}=\sqrt{4{\left(\delta_{d2}{-\delta }_{d1}\right)}^{2}+{\left(\delta_{p2}-\delta_{p1}\right)}^{2}+{\left(\delta_{h2}-\delta_{h1}\right)}^{2}}$$

The solubility increases as the value of *R_a_* decreases towards 0 [34].

#### 2.2.2. Cloud Point Curve Measurement

In order to determine the compatibility of a solvent to fabricate membranes by the non-solvent phase inversion method, a cloud point curve must be obtained for solvent/non-solvent/polymer ternary system [35]. For the PolarClean/PSf/water system, a cloud point curve was experimentally determined by titration [36]. In experiments, dope solutions were prepared using 1, 3, 5, 10, 15, 20, 25 and 30 wt % concentrations of PSf in NMP, PolarClean and DMAc. Each of these dope solutions was mixed using a sonicator (Elmasonic P70H, Elma Electronic Inc., Munich, Germany) at 65 °C (with frequency of 80 kHz, power of 900 W under pulse mode) for 24 h. All dope solutions were then cooled to room temperature, and deionized water was gradually added to the dope solutions using a micropipette until the solutions were observed to become cloudy. Afterwards, the cloudy solution was sonicated for one additional hour to determine if it changed to a clear solution. If the solution was still cloudy, the composition of water/solvent/polymer was determined as the cloud point.

### 2.3. Preparation of PSf Flat Sheet Membranes

The homogeneity of the dope solution is important for the fabrication of membranes [37,38,39]. Two methods were used to achieve full mixing of the dope solution, sonication and planetary mixing. Sonication is a traditional mixing method, while planetary mixing is a 3-D mixing process to mix dry and wet materials using a planetary mixer [40]. The planetary mixing process combines high speed revolution and rotation to accelerate the mixing of polymer/solvent [41], therefore, it has the potential to use in dope solution preparation [42]. 

**Figure 2:**
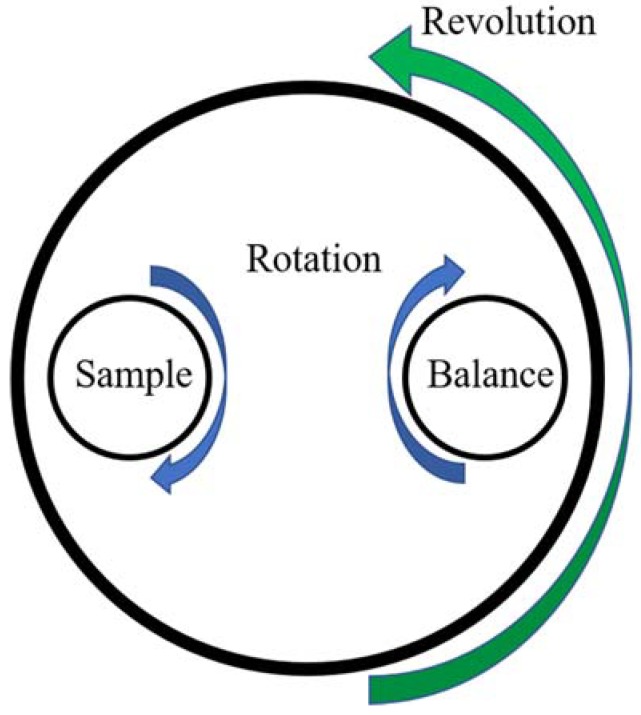
The illustration of the working principle of the planetary centrifugal mixer.

Dope solutions of 17% PSf in NMP, DMAc and PolarClean were prepared using a sonicator (Elmasonic P70H, Elma Electronic Inc., Munich, Germany) and a planetary centrifugal mixer (Mazerustar KK-250S, Kurabo Industries Ltd., Osaka, Japan). The planetary mixer is set up to mimic planetary motion to accelerate the mixing process, where the sample rotates and revolves simultaneously, as shown in Figure 2. The dope solutions were sonicated at 65 °C at a frequency of 80 kHz and power of 900 W under pulse mode for 24 h to accelerate the mixing process, allowed to return to room temperature, and then mixed in the planetary mixer for 10 min. The process was repeated until the solutions became homogenous. After storing in room temperature for three months, the dope solutions did not turn cloudy, which indicated that no separations occurred during this period and the solutions remained homogenous. Room temperature was used for cooling the dope solutions, casting and phase inversion. 

Distilled water was used as the non-solvent. Once the dope solutions were fully dissolved, as determined by the solution becoming clear, the solution was degassed in an ultrasonic bath at room temperature for approximately three hours to remove any air bubbles. After the dope solution was prepared, membranes were cast using the NIPS casting process [43,44,45,46], in which a thin film of the casting solution is deposited onto a glass plate using an aluminum casting knife under room temperature and evaporated for a period between 30 and 120 s in air before being immersed in the non-solvent. Membrane films were cast with thicknesses ranging from 80 to 100 microns. After fabrication, the membranes were stored in deionized (DI) water at room temperature for seven days. 

Numerous membrane treatment methods have been applied to polymeric membranes after casting to achieve desired properties, such as permeability and selectivity [47,48,49]. Some of these include using room temperature ovens, solvent exchange and freeze drying processes to improve membrane performance as measured by flux values [47]. However, to minimize the number of design variables here, only the effect of evaporation time on PSf/PolarClean dope solution prior to casting was investigated using 30, 45, 60, 90 and 120 s. It was determined that evaporation time had a strong effect on water permeability. With increasing evaporation time, the polymer concentration of the polymer-rich phase increases, and a denser and thicker selective layer forms on the surface of the membranes [50]; as a consequence of the increase in selective layer thickness, the permeability of membranes decreases [51,52]. All filtration experiments were performed by first precompacting the membranes using DI water, and then filtering BSA solutions through an Amicon dead-end filtration cell (Amicon Stirred Cell 8010-10 mL, Millipore Sigma company, Burlington, MA, USA) under a constant pressure at 4 bars at room temperature. 

**Figure 3:**
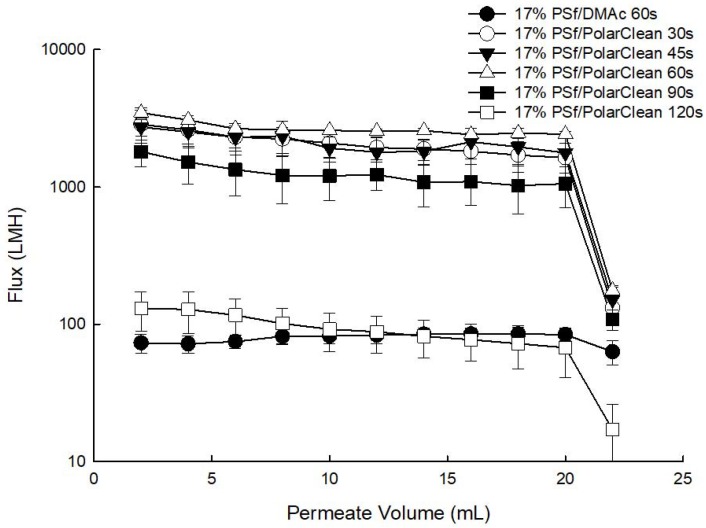
Pure water and BSA solution permeability (at 4 bar).

As shown in Figure 3, after precompaction, water flux values for evaporation times of 30 s (1633 ± 449.3 LMH), 45 s (1769 ± 509.0 LMH) and 60 s (2423 ± 124.9 LMH) were not significantly different. As evaporation time increased from 60 s to 120 s, in agreement with literature studies [50,51,52,53], membrane pure water permeability decreased by over one order of magnitude (Figure 3). Specifically, the water flux value decreased from 2423 ± 124.9 LMH for 60 s to 1055 ± 346.7 LMH for 90 s, and continued decreasing to 67 ± 26.7 LMH for 120 s. Furthermore, at the start of filtration of 1000 ppm BSA feed solution, all membranes displayed declined flux values likely due to instantaneous fouling. For PSf/PolarClean membranes, the initial BSA flux values were 131 ± 41.5 LMH for an evaporation time of 30 s, 151 ± 41.0 LMH for 45 s, 176 ± 8.8 LMH for 60 s, 108 ± 18.7 LMH for 90 s, and 17 ± 9.2 LMH for 120 s. 

For PSf/DMAc membrane, an evaporation time of 60 s was studied, and the associated BSA flux value was 63 ± 12.4 LMH. The probable reason for the significant difference as compared to PSf/PolarClean might be that the selective layers of PSf/PolarClean membranes made with short evaporation times were relatively thinner as compared to PSf/DMAc, as later discussed in Section 3.5. Without a thick selective layer, BSA molecules might have blocked the pores faster, which would lead to instantaneous cake formation, and therefore, a larger decrease the water flux [54,55,56,57,58]. Since 60 s was originally used for DMAc membranes, it was decided to use 60 s evaporation time for PolarClean membranes for a direct comparison.

### 2.4. Characterization of PSf Membranes

#### 2.4.1. Morphology

Flat sheet membranes, prepared using phase inversion method, were immersed and fractured in liquid nitrogen and then sputtered with palladium. The top layers and the cross sections of the PSf membranes were sampled by focused ion beam (FIB) and then observed by SEM (FIB-SEM, FEI Helios Nanolab 660, Thermo Fisher Scientific, Waltham, MA, USA). FIB is an advanced method to sample preparation prior to SEM imaging to characterize the morphology of membranes [59]. FIB-SEM provides a more effective method to observe the cross sections of membranes as compared to the common SEM [60]. Moreover, the higher quality of FIB-SEM images shows the internal structure of polymeric membranes and shows the distribution of pores. FIB-SEM images showed the detailed images at scales of 5 nm. In order to observe the entire morphology of cross sections, the PSf membranes were observed by SEM (SEM, Hitachi S-4300, Hitachi Group, Troy, MI, USA).

#### 2.4.2. Contact Angle

The contact angle data of PSf membranes prepared with different solvents were determined by the sessile drop method using a drop shape analyzer equipped with a high definition camera (DSA100S, Krüss company, Hamburg, Germany). 

#### 2.4.3. Porosity and MWCO

The porosity (Pr, %) of PSf membranes were tested using differences in membrane weight [61,62,63]. Due to the hydrophobicity of PSf, water was not used in this measurement. Membranes were first wet by silwick (Porous Materials Inc., Ithaca, NY, USA), then isopropyl alcohol (IPA, ≥99.7%, FCC, FG, Sigma-Aldrich, St. Louis, MI, USA) was filtered through the membranes under 2 bars for 10 min [63]. The membranes with IPA were exposed to clean and dry air for three days. The wet and dried state weights of membranes at the equilibrium were collected separately [61,62,63,64]. Then the porosity was calculated using the Equation (3):

*Pr* = *V_sil_/V_total_* = (*W_sil_/ρ_sil_)/(W_sil_/ρ_sil_ + W_PSf_/ρ_PSf_*) (3)

where $W_{sil}\;\mathrm{and}\;W_{PSf}$ are the weights of the silwick and PSf membranes; $\rho_{sil}$ is the density of the silwick, 0.93 g/mL and $\rho_{PSf}$ is the density of PSf, 1.24 g/mL [63,65,66].

Molecular weight cut off (MWCO) represents the lowest molecular weight of solute which could be 90% rejected by the membrane [33,67]. Polyethylene glycol (PEG) with molecular weight from 200, 400, 1000, 4000, 10,000, 20,000, 40 kDa were used as feed to determine MWCO values for the different membranes. Since PEGs are linear polymers, the regression model of hydrodynamic radius (nm) and molecular weight (kDa) is given by Equation (4) [68]:

*r_H_*= 0.06127*(MW)*^0.3931^ (4)

Table 2 shows the corresponding hydrodynamic radius (nm) to tested molecular weights of PEGs calculated using Equation (4).

#### 2.4.4. Filtration Studies 

Water permeation experiments were carried out at a constant pressure of 4 bars in an Amicon dead-end filtration cell (Amicon Stirred Cell 8010-10 mL, Millipore Sigma Company, Burlington, MA, USA). The pure water flux was measured using deionized water until the water flux became constant, which is called precompaction. In this study, all the precompaction were performed by filtering 20 mL DI water at a constant pressure of 4 bars for consistency. After precompaction, 20 mL of solutions containing 1000 mg/L BSA; that is, 20 mg of BSA, were filtered through the membranes at a constant pressure of 4 bars and room temperature. BSA has a molecular weight of 66.5 kDa, is a hydrophobic, and its isoelectric point is 4.7, which means it is negatively charged at neutral pH values. The permeability was recorded for every 2-mL filtration period, and permeate samples were collected to analyze the concentrations of BSA in the feed and permeate using a Fusion UV/Persulfate TOC analyzer (14-9600-100, Teledyne Tekmar Company, Mason, OH, USA). All experiments were performed in triplicate.

#### 2.4.5. Recovery and Fouling Performance

After BSA filtration, reverse flow filtration using deionized water was performed at a constant pressure of 2 bars for 30 min to remove foulants that were not adsorbed to the membrane (i.e., reversibly attached), and then flux recovery ratio (Re, %) was measured. The recovery ratio is related to the resistance of the fouled membrane. 

## 3. Results and Discussion

### 3.1. Hansen Solubility Parameter Calculation

RED values of selected solvents (including water as comparison) and PSf are shown in Table 3. The RED value of PolarClean is slightly larger than that of NMP and DMAc; however, a solvent is determined to be a good solvent when the RED value is equal to or smaller than 1 [27]; thus, PolarClean has the potential to fabricate PSf membranes.

### 3.2. Cloud Point Curve

**Figure 4:**
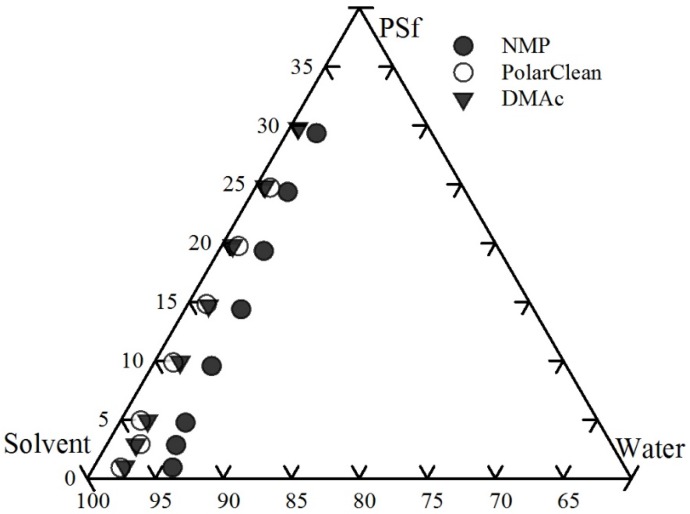
Experimental cloud point curves of PSf/solvent/water system.

The ternary phase diagram is the general method to illustrate the thermodynamics of a polymer/solvent/non-solvent system [25,35,36,69]. The cloud point curve, which is considered as the experimentally binodal curve, represents the composition where the solution is not thermodynamically stable and phase transition occurs. Experimental cloud point curves were developed here for PSf/NMP/H_2_O and PSf/DMAc/H_2_O, and these agreed with literature reported curves [25,69,70]. Thus, the experimental cloud point curve of PSf/PolarClean/H_2_O using the same method was considered valid. The experimental cloud point curves for polymer/solvent/non-solvent are shown in Figure 4. The cloud point curves illustrate that the NMP/PSf solution system had the highest non-solvent (i.e., water) tolerance, while the water tolerance of PolarClean/PSf and DMAc/PSf solutions were not significantly different. Therefore, the miscibility area of the PolarClean/PSf/water and DMAc/PSf/water systems was found to be less than NMP/PSf/water system, while the precipitation rate of PolarClean/PSf and DMAc/PSf solutions was higher than then NMP/PSf solution.

**Figure 5:**
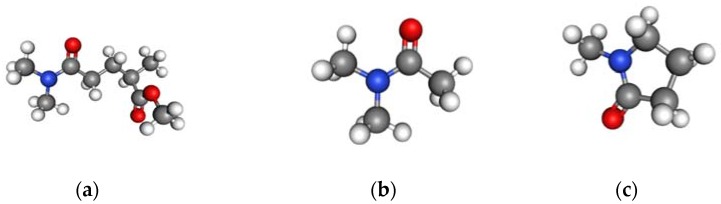
Chemical structure of three solvents: (**a**) PolarClean; (**b**) DMAc; (**c**) NMP. (Blue: Nitrogen, Red: Oxygen, Grey: Carbon, White: Hydrogen).

Moreover, as shown in Figure 5, NMP has a 5-membered ring, which is a distinctly different structure as compared to DMAc and PolarClean since the latter two have similar chain structures, specifically, the structure of PolarClean is a combination of DMAc and isopropyl acetate. Therefore, based on the similar cloud point curves and chemical structures, it was concluded that PSf membranes prepared with PolarClean were best compared to membranes prepared with DMAc for this research.

### 3.3. Porosity and MWCO

**Figure 6:**
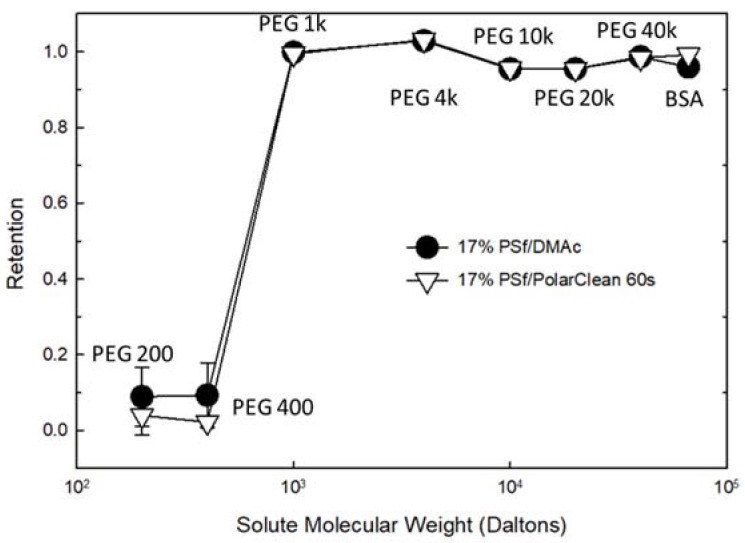
Molecular weight cut-off of PSf membranes.

To determine the types of membranes made here and predict their filtration performance, molecular weight cut-off (MWCO) and porosity studies were performed. Figure 6 showed that there was no significant difference in the MWCO of 17% PSf membranes cast using DMAc and PolarClean, with all the membranes showing rejection to large molecules in the range of 400–1000 Daltons (0.65–0.93 nm of hydrodynamic radius) through the size-exclusion process. The PSf/DMAc membranes showed 68 ± 5.00% overall porosity, while the PSf/PolarClean membranes showed 71 ± 0.77%. As with MWCO, the porosity of these two different membranes showed no significant difference. 

### 3.4. Hydrophobicity of Membranes

Membrane hydrophobicity affects permeability, rejection and fouling behavior of membranes [3,71,72,73,74]. The contact angle of 17% PSf membranes prepared using DMAc was found to be 64 ± 3.52°, while that using PolarClean was 68 ± 2.35°. Therefore, the hydrophobicity of membranes would not account for any differences water permeability, selectivity or fouling observed here, and differences were more likely due to structural differences, as discussed in Section 3.5.

### 3.5. Morphology

**Figure 7:**
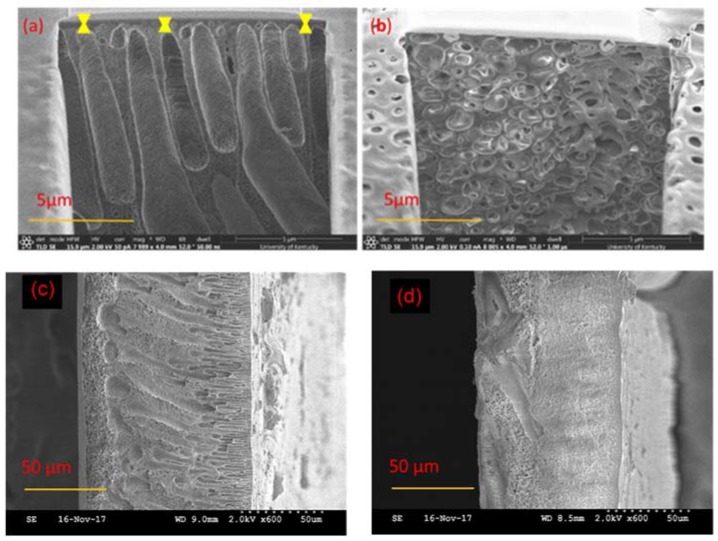
Cross-sectional FIB-SEM images of the top layers of PSf ultrafiltration membranes prepared with: (**a**) DMAc; (**b**) PolarClean. Cross-sectional SEM images of PSf ultrafiltration membranes prepared with: (**c**) DMAc; (**d**) PolarClean.

The cross sections of the selective layers of 17% PSf membranes prepared with the two solvents are shown in Figure 7a,b, and the entire cross sections of the PSf membranes are shown in Figure 7c,d. Figure 7a,b show, at a magnification of 5 μm, the details of the selective layers of the membranes and also the thicknesses of the selective layers, while Figure 7c,d show the entire cross section morphology of the membranes at a magnification of 50 μm. Figure 7a–d show that the PSf membranes prepared with DMAc had finger-like pore structures, whereas membranes prepared with PolarClean had sponge-like pore structures. This difference in morphology is influenced by several factors, including the polymer type, additives, solvent and non-solvent combinations, as well as fabrication techniques [5,75,76]. In NIPS, an instantaneous liquid-liquid demixing process leads to finger-like pore structures, while a delayed liquid-liquid demixing process results in sponge-like pore structures [27,50]. The demixing process happens when the composition profile intersects with the binodal line and the delayed demixing process leads to the slow precipitation, and therefore forms the spongy-like structures [27]. The speed of the liquid-liquid demixing process is determined by the diffusion rate between the solvent and non-solvent. Therefore, from Figure 7c,d, it was hypothesized that the diffusion rate of DMAc and water was faster than that of PolarClean and water. Furthermore, it has been shown that finger-like pores result in higher water fluxes along with lower solute rejection rates as compared to sponge-like pores [77,78,79]. Therefore, it was expected that the membranes prepared using the two different solvents would display differences in permeability.

Another observation was that the thicknesses of the selective layers of the membranes were different. From Figure 7a, PSf/DMAc membranes displayed an approximate selective layer thickness of 400 to 800 nm, while PSf/PolarClean membranes (Figure 7b) did not show an obvious selective layer. The thickness of selective layer is influenced by several factors, including polymer concentration, evaporation time and coagulation bath conditions [50,51,52,53]. In this case, it is proposed that evaporation time might have been the most probable cause of the difference in thicknesses. Since different solvents have different volatilities, the evaporation time to form the selective layers with same thicknesses may differ significantly. If the boiling point is used to represent the volatility [80], for a given solvent, the higher the vapor pressure and the lower boiling point, the higher volatility [80]. The vapor pressure at 20 °C is 300 Pa for DMAc [81] and less than 0.01 Pa for PolarClean [82]. The boiling points at 101.3 kPa of DMAc and PolarClean are reported to be 165 °C and 282 °C [21,83], respectively, which indicated that DMAc had higher volatility than PolarClean. Therefore, a longer evaporation time would be needed to form the same thickness of selective layer for PSf/PolarClean membranes as PSf/DMAc membranes, which agrees with experimental effects of evaporation time (Figure 3). From Figure 3, an evaporation time of 120 s for PSf/PolarClean led to membranes of similar permeability as an evaporation time of 60 s for PSf/DMAc. 

### 3.6. Water Flux

**Figure 8:**
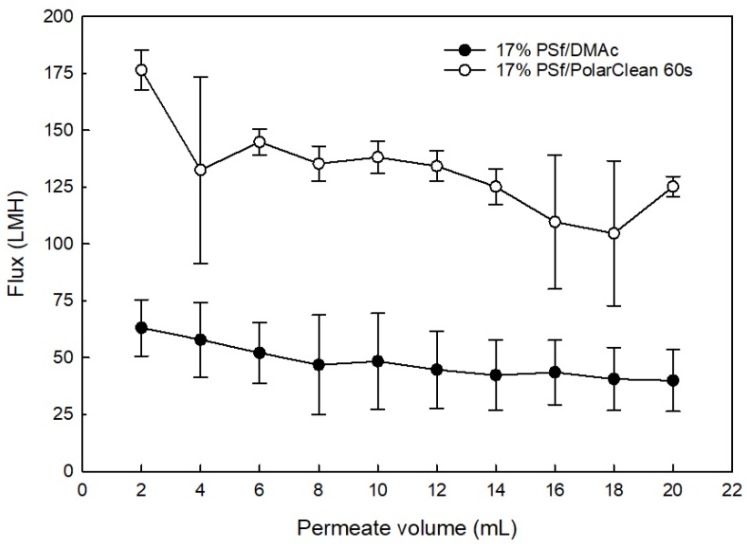
BSA solution permeability (at 4 bar).

From Figure 8, during BSA filtration, the flux of PSf/DMAc membranes declined from 63.1 ± 12.4 to 40.0 ± 13.6 L/m^2^·h (LMH), while the flux of PSf/PolarClean membranes declined from 176.0 ± 8.8 to 125.0 ± 4.5 LMH. This decline was likely due some compression under the 4-bar pressure [84,85] along with accumulation of BSA on the surface of the membrane, which increased resistance to flow. It was also observed that the water flux values of PSf/PolarClean membranes were higher than PSf/DMAc membranes, which did not agree the expectation from the morphology (Figure 7). However, as previously mentioned, for different solvents, the evaporation time impacted the thickness of the selective layer of the membranes, which consequently impacted the water permeability [52,53,86], which may explain the higher flux values observed with PSf/PolarClean membranes. In this case, the thickness of the selective layers of membranes were the dominant parameter influencing water permeability. 

BSA rejection analysis was also performed, and it was determined that 99 ± 0.51% of BSA was removed by the PSf/PolarClean membranes, which was slightly higher than 96 ± 2.00% BSA rejection by the PSf/DMAc membranes. The higher rejection rate of PSf/PolarClean membranes agreed their sponge-like pore structures as shown in Figure 7b. In addition to comparing the performance of PSf/PolarClean membranes fabricated here to the performance of PSf/DMAc membranes fabricated using similar controlled conditions, a comparison was made to literature PSf/DMAc membranes [2,87,88,89,90], and is shown in Table 4. It is important to note that evaporation times were not available for membranes from the literature. From Table 4, it is clearly observed that PSf/PolarClean membranes are within the acceptable range of pure water flux, permeability and BSA rejection. This validates the potential of PolarClean to be used as a bio-derived greener alternative equivalent to DMAc. 

**Figure 9:**
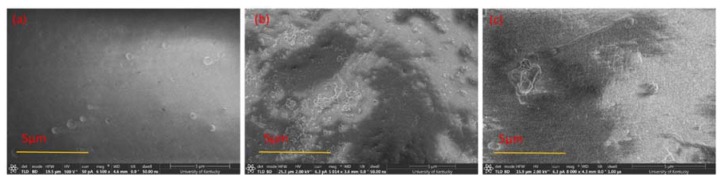
FIB-SEM images of PSf/PolarClean membranes surface: (**a**) Original; (**b**) After BSA filtration; (**c**) After DI water backwash.

**Figure 10:**
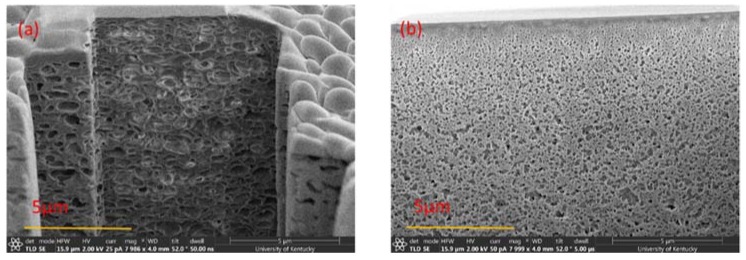
Cross-sectional FIB-SEM images of PSf/PolarClean ultrafiltration membranes: (**a**) After BSA filtration; (**b**) After DI water backwash.

To determine the extent of the irreversible adsorption of BSA to the membranes after filtration, or the amount of irreversible fouling, reverse-flow filtration using DI water was performed after BSA filtration to represent a backwash cycle. The flux was then measured after reverse-flow filtration to determine the recovered flux, or the percentage of the flux decline due to reversible fouling. The average recovered flux for PSf/PolarClean membranes was 30 ± 0.88%, which was lower than that for PSf/DMAc membranes (77 ± 13.28%). The lower flux recoveries using PolarClean was hypothesized to have been due to the sponge-like pores collapsing after filtration, which would make it more difficult for water to pass through the pores. In order to verify this hypothesis, PSf/PolarClean membranes were analyzed using FIB after BSA filtration and reverse-flow filtration. Figure 9 shows the surface morphology, while Figure 10 displays the cross-sectional structures. As observed from Figure 9b, BSA molecules accumulated on the surface of the membranes to foul them, and reverse-flow filtration was able to remove most of the foulants (Figure 9c). However, comparing Figure 7b (cross-section using PolarClean) and Figure 10a (cross-section using PolarClean after BSA filtration) shows that the pores of the PSf/PolarClean membranes slightly collapsed after BSA filtration, which might have been due to the pressure applied on the membranes [91,92,93]. Likewise, by comparing Figure 10a (cross-section using PolarClean after BSA filtration) and Figure 10b (cross-section using PolarClean after backwash), it was observed that the pores collapsed further and the thickness of the selective layer increased after backwash. This was likely the cause of the lower recovery rate observed for PSf/PolarClean membranes. One reason for the collapse of the pores might have been that the mechanical strength of the sponge-like pores was lower than finger-like pores; hence, the sponge-like pores might have been easier to compress under the same backwash pressure. 

Another reason might be the selective layer of the PSf/PolarClean membranes were thin, in agreement with Figure 7b, and possibly fragile. Considering that the MWCO of PSf/PolarClean membranes was less than 1 nm (Figure 6) while the apparent the pore size of PSf/PolarClean membranes was at micrometer level (from SEM images, Figure 7b, a thin selective layer might have been present. This selective layer might have collapsed or been damaged during filtration and/or recovery. Then, the selectivity of the membrane became a function of the porous structure and tortuosity, indicating it started to behave as a depth filter instead of a membrane. This also correlates with the low recovery following backwashing, indicating that the pores collapsed. 

## 4. Conclusions

In this study, PolarClean was used as a NIPS solvent to cast PSf membranes and then compared with DMAc. Based on the ternary phase diagram, the cloud point curve of PSf/PolarClean/water was similar to that of PSf/DMAc/water. Dope solutions for PSf/PolarClean and PSf/DMAc were prepared at 65 °C and membranes were cast and characterized afterwards. The overall porosity, MWCO and hydrophobicity of membranes made using PolarClean and DMAc were not significantly different. However, the cross-sections images of the membranes were different, with PSf/DMAc membranes showing finger-like structures and PSf/PolarClean membranes showing sponge-like structures. Regarding membranes performance, PSf/PolarClean membranes showed slightly higher BSA rejection rates (99 ± 0.51%) as compared to PSf/DMAc membranes (96 ± 2.00%), which agreed with their sponge-like pores structures. Furthermore, PSf/PolarClean membranes also showed higher flux values (176.0 ± 8.8 LMH) than PSf/DMAc membranes (63.1 ± 12.4 LMH), which disagreed the sponge-like structure theory and might have been due to evaporation time. However, pore collapsing was observed in the study, which means the long-term stability of PolarClean membranes are uncertain. In conclusion, bio-derived solvents should be investigated further and may become promising replacements to traditional solvents. 

## Figures and Tables

**Table 1:** Physicochemical properties of NMP, DMAc and PolarClean.

Properties	NMP	DMAc	PolarClean
CAS-No	872-50-4	127-19-5	1174627-68-9
Formula	C5H9NO	C4H9NO	C9H17NO3
MW (g·mol^−1^)	99.133	87.122	187.239
Density (g·mL^−1^)	1.03	0.94	1.043
Flash point (°C)	95	69	144–146
Boiling point (°C)	202	165	278–282
Solubility in water (%)	miscible	miscible	miscible
Signal	Danger	Danger	Warning
Toxicity	Reproductive toxicity	Reproductive toxicity	

**Table 2:** Molecular weights and hydrodynamic radius of PEG used in MWCO study.

Molecular Weight (kDa)	Hydrodynamic Radius (nm)
PEG 200	0.49
PEG 400	0.65
PEG 1000	0.93
PEG 4000	1.60
PEG 10,000	2.29
PEG 20,000	3.01
PEG 40,000	3.95

**Table 3:** Relative energy density calculation for picked solvents and PSf.

Polymer	$\delta_{d}$ (MPa1/2)	$\delta_{p}$ (MPa1/2)	$\delta_{h}$ (MPa1/2)	Ro (MPa1/2)	
PSf	19.7	8.3	8.3	8.00	
Solvents	$\delta_{d}$ **(MPa1/2)**	$\delta_{p}$ **(MPa1/2)**	$\delta_{h}$ **(MPa1/2)**	**Ra (MPa1/2)**	**RED**
NMP	18	12.3	7.2	5.36	0.67
DMAc	16.8	11.5	10.2	6.89	0.86
PolarClean	15.8	10.7	9.2	8.21	1.03
Water	15.5	16	42.4	35.95	4.49

**Table 4:** Comparison between PSf/PolarClean membranes and literature PSf/DMAc membranes.

Polymer	Solvent	Polymer (wt %)	Pressure (Bar)	Pure Water Flux (LMH)	Pure Water Permeability (LMH per Bar)	Feed Solution	Rejection	Source
PSf	PolarClean	17	4	2423	605.75	1 g/L BSA	99%	This study
PSf	DMAc	17	4	63	15.75	1 g/L BSA	96%	This study
PSf	DMAc	16	4	21	5.25	milk	98% of protein	[90]
PSf	DMAc	18	4	36.7	9.175	unreported	unreported	[91]
PSf	DMAc	17.5	4	41.2	10.3	1 g/L BSA	90%	[92]
PSf	unreported	-	4	133	33.25	2.5 g/L Dextran (MW 18 k)	88.80%	[93]
PSf	unreported	-	3.8	1500–2400	394.74–631.58	10 kDa	90%	[2]
